# Genetic Structure of a Naturally Regenerating Post-Fire Seedling Population: *Pinus halepensis* As a Case Study

**DOI:** 10.3389/fpls.2016.00549

**Published:** 2016-04-27

**Authors:** Anna Gershberg, Gidi Ne'eman, Rachel Ben-Shlomo

**Affiliations:** ^1^Department of Evolutionary and Environmental Biology, Faculty of Natural Sciences, University of HaifaHaifa, Israel; ^2^Department of Biology and Environment, Faculty of Natural Sciences, University of Haifa-OranimTivon, Israel

**Keywords:** disturbance, fire, natural selection, pine, spatial demographic structure, spatial genetic structure

## Abstract

To study the effects of wildfire on population genetics of a wind pollinated and wind dispersed tree, we have analyzed the genetic structure of a post-fire, naturally regenerating seedling population of *Pinus halepensis* Miller, on Mt. Carmel, Israel. We tested the existence of spatial genetic structure, which is expected due to the special spatial demographic structure of the post-fire seedling and sapling populations of this species. Explicitly, we asked whether or not seedlings that germinated under large, burned, dead pine trees are also their offspring. The results revealed that the post-fire seedling population is polymorphic, diverse, and reflects the pre-fire random mating system. In contrast to our prediction, we found no division of the post-fire seedling population to distinct sub-populations. Furthermore, as a result of post-fire seed dispersal to longer range than the average pre-fire inter-tree distance, seedlings found under individual burned trees were not necessarily their sole offspring. Although the population as a whole showed a Hardy-Weinberg equilibrium, significant excess of heterozygotes was found within each tallest seedlings group growing under single, large, burned pine trees. Our finding indicates the possible existence of intense natural selection for the most vigorous heterozygous genotypes that are best adapted to the special post-fire regeneration niche, which is the thick ash bed under large, dead, pine trees.

## Introduction

Wildfires are a recurring disturbance in the Mediterranean basin, and a major factor affecting the ecology and evolution of plants in Mediterranean-type ecosystems (Naveh, 1994; Keeley et al., 2011). More than 10^6^ ha of forests were consumed by fires in the southern EU during 1980–2000 (European Commission, 2001). In Israel, about 13,600 ha of natural maquis and planted pine forests were consumed by fire during 1980–1991 (Kleiot and Keider, 1992); in December 2010, one fire consumed about 2500 ha covered mainly by pine forests (Perevolotsky et al., 2011).

*Pinus halepensis* Miller (Aleppo pine) is a main constituent of Mediterranean lowland forests (Barbéro et al., 1998; Quézel, 2000). It is primarily a western Mediterranean tree (Mirov, 1967), with some small, native and disjunct populations in Israel (Barbéro et al., 1998). The Israeli natural populations of *P. halepensis* differ in their genetic composition from all the other native populations, and were defined as the eastern variety (Korol et al., 2002).

Aleppo pine is a highly flammable tree that is killed by fires, consequently it is an obligate seeder whose post-fire populations depend solely upon seed germination (Ne'eman and Trabaud, 2000; Pausas, 2015). Post-fire germination of pine seeds consists mainly of seeds originating from serotinous cones that comprise a canopy stored seed bank, which release their seeds primarily after crown fires (Lamont et al., 1991; Nathan et al., 1999). Young *P. halepensis* trees have larger proportion of serotinous cones than older trees, which reduced the risk of being burned before establishing a large enough canopy stored seed bank (juvenility risk); and post-fire established populations have also higher degree of serotiny than those established in the absence of fire (Ne'eman et al., 2004). Aleppo pine populations that face repeated fire episodes showed finer-scale spatial aggregation of serotiny relative to those residing lower fire recurrences areas (Hernandez-Serrano et al., 2013). Seeds from serotinous cones are better adapted for post-fire germination than those released from regular cones (Goubitz et al., 2003).

The fine scale spatial distribution of large Aleppo pine trees that are burned by canopy fires determine the spatial structure of the post-fire pine seedlings generation. The extremely low pH of the thick ash layer, deposited during the fire under burned pine canopies, inhibits the germination of many herbaceous and woody species but less so of Aleppo pine seeds (Henig-Sever et al., 1996; Ne'eman and Izhaki, 1999; Eshel et al., 2000; Izhaki et al., 2000). Consequently, pine seedlings that grow under large burned pine canopies experience lower inter-specific competition, grow faster, and have a higher probability to comprise the post-fire pine forest generation than those growing elsewhere. Therefore, the spatial demographic pattern of post-fire forest is similar to the pre-fire forest (Izhaki et al., 1992; Ne'eman et al., 1995; Ne'eman and Izhaki, 1998; Nathan and Ne'eman, 2004).

Most (97%) of *P. halepensis* seeds are dispersed by wind over relatively short distances of up to 20 m from their mother trees, and 72% fall under their canopies (Nathan, 1999; Nathan et al., 2000; Nathan and Ne'eman, 2000). The pre- and post-fire demographic patterns are comparable. In the absence of data regarding post-fire seed dispersal, and under the assumption that the pattern is similar to dispersal with no fire, we can hypothesize that most of the post-fire dispersed seeds find their preferred regeneration niche under the canopy of their mother trees.

Pines reproduce only sexually via seed germination. Conifers including pines have not been reported for self-incompatibility (Hagman, 1975). Facultative selfing was found in pines (Reviewed by Ledig, 1998): medium inbreeding levels were reported for *P. pinaster* (De-Lucas et al., 2009), low levels for *P. strobus* (Marquardt and Epperson, 2004) and no inbreeding was found in *P. brutia* (Panetsos et al., 1998).

Fine scale spatial genetic structure (FSSGS) is the nonrandom spatial distribution of genotypes and alleles, which commonly results from fine-scale aggregation of siblings within a population (Wells and Young, 2002; Vekemans and Hardy, 2004). FSSGS of reproductive *P. halepensis* individual trees was empirically studied over time in an expanding native population; in early stages the genotypes were randomly distributed in space, but over time, FSSGS developed through increased genetic clustering with increasing density (Troupin et al., 2006). FSSGS was explained by fine-scale environmental heterogeneity and possibly by micro-environmental selection, inbreeding, and individual variation in the reproductive success of trees (Troupin et al., 2006).

Shohami and Nathan (2014), studying recently Mt. Carmel *P. halepensis* population (the same area as in this study), reported a limited pre-fire pollen dispersal and consequently, significant genetic structure and high kinship. Hence, the post-fire open landscape permits pollen gene flow over greater distances.

Disentangling the effects of fire on spatial genetic structure of post-fire regenerating seedling populations is an important challenge that has not yet been addressed. In this research we aimed to study the effects of fire on the genetic composition, variability and spatial genetic structure of the first naturally regenerating post-fire seedling population of *P. halepensis*, as a model for trees with wind dispersed seeds and pollen. Specifically we: (1) examined the genetic structure and variability of the first regenerating post-fire pine seedling population and compared it to near non-burned adult pine stands; (2) checked whether there was a spatial genetic structure in this regenerating seedling population, and (3) determined whether the tallest seedlings growing under large burned trees are also their descendant siblings. We hypothesized that: (1) The genetic structure and variability of the first post-fire seedling population is similar to that of unburned populations in the same geographical region (Mt. Carmel, Israel). (2) There is a spatial genetic structure in the regenerating post-fire seedlings population; namely, the variation within the seedlings groups growing under a dead tree will be smaller than that among the seedling groups growing under different dead trees. (3) The seedlings growing under a burned tree are also its descendant siblings.

## Methods

### Study site

The study site was located within a native *P. halepensis* forest area in Lubim on Mt. Carmel, Israel (E35°00′ N32°44′). This site was an abandoned field that has been colonized by native pines about 60 years ago. The climate is typical east Mediterranean one with mean annual rainfall of 700 mm that falls mainly from December to February, and a long, hot and dry summer from May to October. The forest was completely burned (i.e., all the large mature trees did not survive the fire) in autumn 2006 and was left for natural regeneration. Prior to the fire in 2006, the multi-ages stand (ca 140 ha), was comprised of about 150 large trees; in a distance of more than 1Km from any other pine population. The site has not been under fire prior to 2006.

### Sampling method

**Figure 1:**
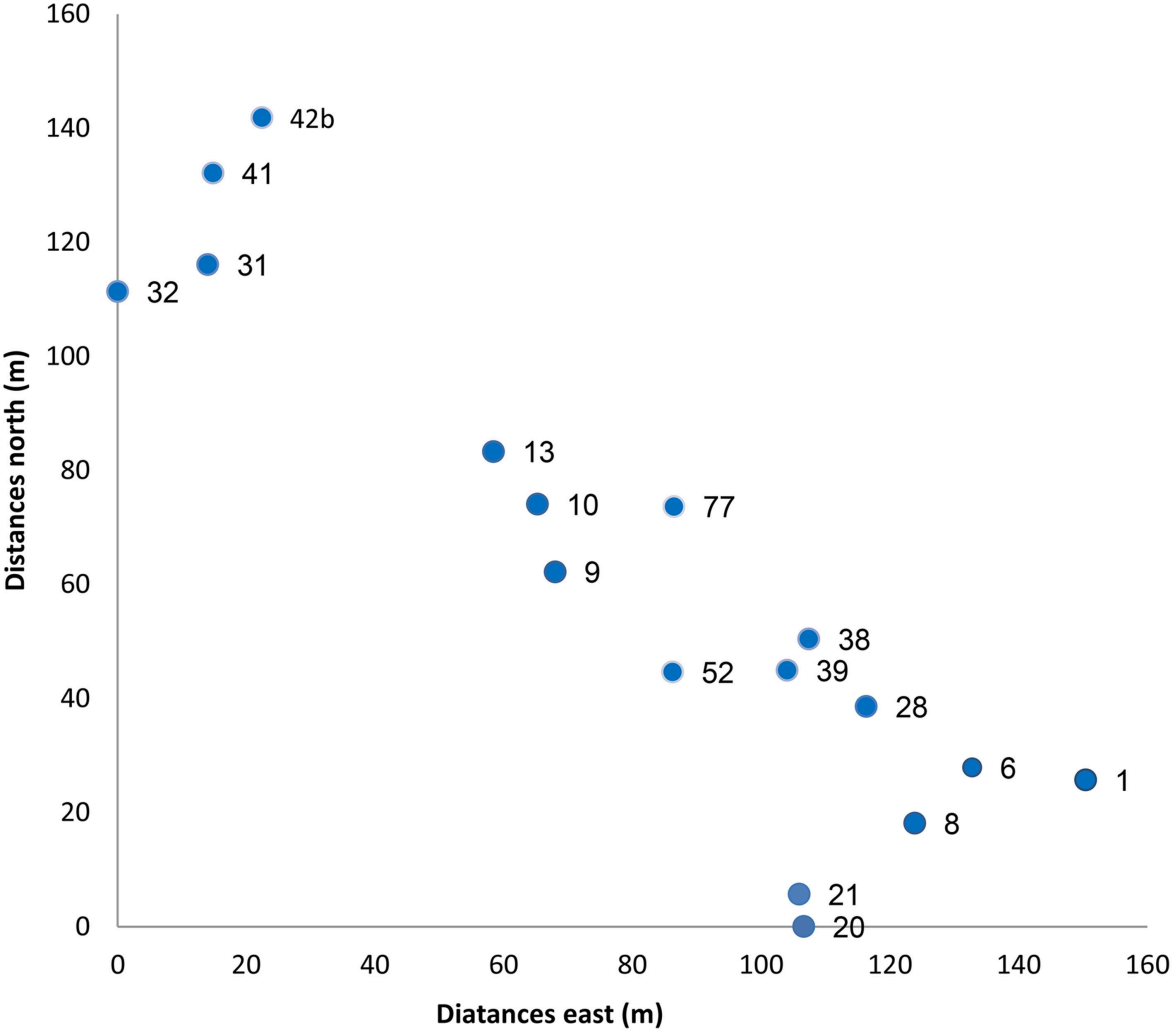
**Spatial location of the sampled trees (distances are in meters)**. The numbers next to each dot represent the number of the burned trees and their seedling groups.

After the 2006 fire, within the 140 ha site, we have selected, numbered and mapped 20 large dead trees with canopy diameter of 5 to 10 m. The canopies of the chosen trees were at least 6 m apart (up to 18 m between any two adjacent trees, average 13 m between neighboring trees; Figure 1). In the spring 2008, one and a half years after the fire, we sampled the 10 tallest seedlings (out of tens to several hundreds) growing up to 2 m radius around the trunks of all selected burned trees. This non-random selection was done as we wanted to sample the seedlings that had the highest probability to replace the burned tree. These groups of 10 seedlings under any given tree will be referred hereafter as a seedling “group”—the number of each group is that of the burned tree. Ten to fifteen fresh needles were sampled from each seedling in all groups and were kept separately in an icebox until transferred to the lab and stored at −20°C.

### Molecular analysis

Frozen needles (20–80 mg) were crushed to powder using the TissueLyser device (QIAGEN). Lysis buffer AP1/RTL (QIAGEN) was added to the samples. DNA was extracted from the lysate using Biosprint 15 (QIAGEN; following BS15 DNA PLANT protocol). Nine known nuclear microsatellite loci were amplified by PCR (Table 1S; Keys et al., 2000; Mariette et al., 2001; González-Martínez et al., 2004; Guevara et al., 2005; Steinitz et al., 2011, 2012a). Each 15 μl PCR reaction contained template DNA (~20 ng), 0.2 μM of each of the primers and 7.5 μl enzyme Taq Master Mix Purple 2x (Lamda Biotech, St. Louis, Missouri, USA). The forward primer of each microsatellite was labeled with florescent dye (6-Fam, Vic, Ned or Pet). To validate the integrity of amplification, we repeated genotyping for 10–20% of the samples for each locus.

Amplification products were separated using ABI 3130xl Florescence-Reader (Applied Biosystems). Manual scoring of PCR product size with reference to a 500-Liz standard marker (Applied Biosystems) was made using peak scanner version 1.0 (Applied Biosystems) and GeneMapper software version 4 (Applied Biosystems).

### Data analysis

Unfortunately, we failed to amplify DNA from the burned trees, and thus could not genetically type them. Therefore, to estimate the minimal possible numbers of mothers for each seedling group, we compared the genetic structure of seedlings within each group, using the locus ITPH4516, which displayed 15 different alleles within the studied population. If a seedling group shared a mutual heterozygote mother tree, each seedling within the group should show either one of the two possible alleles in the ITPH4516 locus (e.g., if a mother is heterozygote A_1_A_2_ in a locus, all her offspring should comprise of either A_1_ or A_2_). If a group of seedlings presented more than such two mutual alleles, they should share more than one founder. Additional alleles can be contributed by various fathers. This test cannot confirm our “each group one mother” hypothesis, but it can negate it, if many seedling groups show a minimum of more than one mother that had contributed to their genetic structure.

To determine whether the sampling, which was not random, represents a homogenous population, we performed three virtual random sampling sessions of small data sets (*N* = 20) from the full data set (*N* = 181). We used GenAlEx 6.41 (Peakall and Smouse, 2006) to calculate genetic distance (D; Nei, 1978), as well as the genetic differentiation (*F*_ST_ based AMOVA) of the three sets and the whole data (altogether 4 sets). The four data sets did not differ from each other (*D* = 0.0; *F*_ST_ = 0; *p* = 0.4). Therefore, we considered our data set as representing the whole Lubim population. To substantiate that the Lubim population truly represents the Carmel native pine populations, we compared gene diversity parameters of Lubim to three additional populations (Mitla, burned in 1983, Antenna and Beit Oren (Figure 1S) with no evidence of previous fires). In each population we sampled minimum 30 large trees, at least 5 m apart. These four populations demonstrated similar gene diversity levels (Table 2S).

We scored the level of observed heterozygosity (Ho) and calculated the level of unbiased expected heterozygosity (He), inbreeding coefficient (*F*_IS_), Hardy-Weinberg equilibrium (HWE), and genetic distance (D), for the whole Lubim seedling population and separately for each seedling group. Data were analyzed by Tools for Population Genetic Analyses (TFPGA) V 1.3 (Miller, 1997) and by GenAlEx V 6.41 (Peakall and Smouse, 2006) softwares.

The significance level of seedling groups' differentiation (pairwise analysis of all groups; Exact tests—Raymond and Rousset, 1995) based on genetic distance between the groups (Nei, 1978), was determined after 1000 dememorization steps and 10 batches of 2000 permutations per batch, using TFPGA software version 1.3 (Miller, 1997). Bonferroni correction for significance level for that test was extremely strict (*p* < 0.0003). Considering the controversial use of Bonferroni correction (see Field, 2005), we also used a less stringent value for multiple test of *p* < 0.005. When using this significance level, less than 1 of 190 comparisons will show significant genetic difference by chance alone.

The correlation between genetic and geographical distances was tested by Mantel test, using GenAlEx6.41 software (Peakall and Smouse, 2006). The degree of the genetic difference between the seedlings within each group and among the groups was also tested by molecular analysis of variance (AMOVA) based on *F*_ST_ values (Wright, 1965) following Michalakis and Excoffier (1996; 999 permutations).

We also applied a Bayesian clustering method (STRUCTURE) that divided the samples into possible homogenous groups (sub populations) according to their degree of similarity (Pritchard et al., 2000, 2010). We checked clustering possibilities for *K* values from 1 to 20 (STRUCTURE 2.3.4 admixture model; LOCPRIOR option; burn-in of 100,000 steps and 100,000 iterations). The inference of the probable number of clusters was extracted by the log likelihood for each putative number of populations (K; four replicates for each K), Ln P(D) = L(K), and by the delta K method (Evanno et al., 2005), using the program Structure Harvester (Earl and vonHoldt, 2012).

To further compare genetic similarity of seedlings within and between groups, we selected the individuals that had genotypic data for minimum 8 loci (78 seedlings in total) for supplementary cluster analysis (UPGMA). The UPGMA cluster analysis assumed equal evolutionary rate along all branches which considered suitable for comparing regenerating seedlings groups within a population. Each of the 20 seedling groups had at least one individual in this analysis, and 17 groups had at least two seedlings.

## Results

### The genetic structure of the seedling groups

All the analyzed microsatellite loci were polymorphic, ranging from two to 15 alleles, with an average (± SE) of 2.5 ± 0.1 alleles per locus per seedling group (Table 1). The observed heterozygosity (Ho) of the population (±SE) was 0.450 ± 0.080, and the unbiased expected heterozygosity (He) was 0.444 ± 0.0079. Fixation indices indicated that while inbreeding coefficient within individuals relative to the group (*F*_IS_) were generally negative (i.e., excess of heterozygotes); genotypic frequency within individuals relative to the population (*F*_IT_) was positive and close to 0 (0.017 ± 0.043; Table 3S). All nine loci exhibited Hardy-Weinberg equilibrium (HWE; Table 4S). Nonetheless, most of the post-fire seedling groups (16 out of 20) indicated within group negative *F*_IS_ (i.e., excess of heterozygotes (Table 1); this situation is significantly different from random (*p* = 0.0118, two-tail Sign test). Heterozygote excess was observed for all microsatellites within each group (108 out of 147; *p* < 0.0001, two-tail Sign test; Table 5S).

**Table 1:** **Genetic diversity of the 20 post fire seedling groups (each growing under different burned dead pine trees)**.

**Group**	***N***	**Na**	**P (%)**	**Ho**	**He**	***F*_*IS*_**
1	8	2.333 ± 0.645	78	0.440 ± 0.116	0.373 ± 0.097	−0.303
4	9	2.667 ± 0.471	89	0.459 ± 0.117	0.441 ± 0.095	−0.119
6	10	2.556 ± 0.377	89	0.504 ± 0.101	0.460 ± 0.079	−0.174
8	9	2.778 ± 0.596	89	0.535 ± 0.107	0.462 ± 0.093	−0.278
9	10	2.667 ± 0.577	89	0.437 ± 0.086	0.479 ± 0.080	−0.008
10	10	2.778 ± 0.465	89	0.392 ± 0.077	0.438 ± 0.084	0.023
13	10	2.000 ± 0.289	78	0.384 ± 0.108	0.333 ± 0.089	−0.224
15	10	2.222 ± 0.324	78	0.415 ± 0.092	0.426 ± 0.083	−0.057
20	10	2.889 ± 0.716	78	0.539 ± 0.129	0.430 ± 0.100	−0.324
21	10	2.556 ± 0.412	89	0.440 ± 0.093	0.421 ± 0.084	−0.123
28	7	1.667 ± 0.289	44	0.252 ± 0.131	0.272 ± 0.109	−0.043
31	8	2.444 ± 0.503	78	0.328 ± 0.114	0.374 ± 0.102	0.007
32	8	2.444 ± 0.503	78	0.449 ± 0.112	0.396 ± 0.096	−0.216
38	10	2.889 ± 0.512	89	0.486 ± 0.101	0.454 ± 0.081	−0.135
39	6	2.222 ± 0.324	78	0.574 ± 0.137	0.467 ± 0.104	−0.484
41	9	2.778 ± 0.547	100	0.453 ± 0.107	0.423 ± 0.091	−0.116
42	9	2.222 ± 0.324	78	0.405 ± 0.097	0.366 ± 0.085	−0.219
52	9	2.778 ± 0.572	89	0.418 ± 0.092	0.437 ± 0.081	0.001
77	9	2.333 ± 0.408	78	0.357 ± 0.104	0.387 ± 0.092	0.000
42b	10	2.111 ± 0.261	78	0.393 ± 0.094	0.347 ± 0.082	−0.204
Total			100	0.450 ± 0.080	0.444 ± 0.079	−0.149 ± 0.028

*N, Sample size; Na, Average number of alleles per locus (±SE); P, polymorphism (% polymorphic loci); Ho, Observed heterozygosity (±SE); He, Unbiased expected heterozygosity (±SE); F_IS_, inbreeding coefficient*.

The locus ITPH4516 exhibited the highest number of alleles (15), of which the most frequent were of 158 bp (29.6%) and 140 bp (26.2%). Eighteen of the 20 seedling groups presented four or more alleles in this locus, allowing assessment of maternal contribution. At least 11 seedlings groups, each growing under different burned pine tree, were comprised of seedlings that originated from two or more different maternal trees (Table 6S).

### Genetic differentiation among groups

The genetic distances among the seedling groups were small ranging from 0.000 to 0.243 (Table 2). No significant pairwise differences between groups were detected by Exact test using strict Bonferroni correction of α < 0.0003; and only three significant pairwise differences between groups when less strict α < 0.005 (Table 2; lower-left triangle). Similarly genetic differentiation among the tested group was relatively low (*F*_ST_ = 0.144 ± 0.032, and the number of migrants between group, Nm = 1.786 ± 0.243; Table 7S). Mantel test indicated no correlation between geographic and genetic distances (*r*_xy_ = 0.075; *p* = 0.32; *R*^2^ = 0.0056; *y* = 0.0001x + 0.0625). Molecular analysis of variance (AMOVA) indicated that most of the variance (95%) was found within groups and only 5% originated from differences among groups (Table 3).

**Table 2:** **Genetic distances (unbiased) between group pairs (upper-right triangle), and the Exact test significane levels of the pairwise genetic difference (lower-left triangle); three cases of significant differences (*p* < 0.005) are presented in bold font**.

	**1**	**4**	**6**	**8**	**9**	**10**	**13**	**15**	**20**	**21**	**28**	**31**	**32**	**38**	**39**	**41**	**42**	**52**	**77**	**42b**
1	^*****^	0.186	0.174	0.125	0.171	0.180	0.194	0.194	0.179	0.199	0.262	0.182	0.204	0.161	0.126	0.202	0.213	0.189	0.180	0.182
4	0.887	^*****^	0.000	0.000	0.055	0.061	0.045	0.010	0.085	0.071	0.255	0.048	0.000	0.026	0.000	0.045	0.121	0.030	0.000	0.186
6	0.909	0.946	^*****^	0.000	0.034	0.030	0.033	0.000	0.022	0.012	0.150	0.049	0.002	0.008	0.000	0.023	0.067	0.008	0.000	0.128
8	0.099	0.975	0.982	^*****^	0.014	0.018	0.008	0.000	0.000	0.000	0.150	0.032	0.000	0.000	0.000	0.022	0.042	0.005	0.000	0.102
9	0.139	0.570	0.439	0.458	^*****^	0.000	0.017	0.028	0.034	0.063	0.102	0.000	0.036	0.010	0.000	0.034	0.013	0.035	0.075	0.023
10	0.241	0.304	0.404	0.375	0.981	^*****^	0.000	0.018	0.028	0.075	0.108	0.002	0.027	0.000	0.000	0.074	0.000	0.082	0.071	0.045
13	0.326	0.135	0.276	0.677	0.467	0.989	^*****^	0.022	0.059	0.109	0.168	0.046	0.013	0.012	0.024	0.128	0.000	0.107	0.054	0.146
15	0.300	0.848	0.998	0.930	0.438	0.646	0.651	^*****^	0.041	0.036	0.088	0.061	0.001	0.024	0.000	0.032	0.034	0.061	0.009	0.093
20	0.264	0.058	0.554	0.937	0.301	0.306	0.258	0.472	^*****^	0.000	0.177	0.059	0.064	0.055	0.019	0.066	0.035	0.058	0.062	0.070
21	0.080	0.107	0.442	0.931	0.007	0.009	0.007	0.303	0.875	^*****^	0.153	0.065	0.104	0.082	0.000	0.022	0.115	0.017	0.067	0.105
28	0.745	0.847	0.895	0.865	0.905	0.895	0.338	0.945	0.628	0.522	^*****^	0.115	0.279	0.107	0.074	0.068	0.110	0.197	0.243	0.095
31	0.061	0.430	0.125	0.357	0.690	0.563	0.206	0.110	0.251	0.024	0.836	^*****^	0.093	0.017	0.000	0.028	0.052	0.068	0.099	0.075
32	0.677	0.753	0.903	0.819	0.400	0.713	0.820	0.976	0.179	**0.004**	0.038	0.019	^*****^	0.035	0.010	0.116	0.055	0.087	0.000	0.148
38	0.689	0.425	0.631	0.697	0.320	0.815	0.546	0.510	0.143	0.039	0.957	0.278	0.322	^*****^	0.000	0.061	0.014	0.083	0.031	0.097
39	0.841	1.000	0.926	0.771	0.725	0.864	0.226	0.763	0.300	0.416	0.751	0.871	0.464	0.750	^*****^	0.000	0.033	0.007	0.010	0.000
41	0.151	0.748	0.481	0.475	0.203	0.045	**0.002**	0.468	0.142	0.446	0.871	0.262	0.014	0.109	0.744	^*****^	0.135	0.033	0.068	0.090
42	0.262	0.067	0.138	0.657	0.546	0.996	0.996	0.648	0.635	0.010	0.819	0.159	0.578	0.817	0.293	0.008	^*****^	0.157	0.113	0.079
52	0.158	0.468	0.759	0.572	0.287	0.029	0.018	0.226	0.045	0.327	0.385	0.058	0.015	0.014	0.657	0.423	**0.001**	^*****^	0.043	0.115
77	0.992	0.982	1.000	0.948	0.411	0.330	0.112	0.967	0.308	0.065	0.893	0.065	0.887	0.712	0.887	0.508	0.210	0.492	^*****^	0.175
42b	0.849	0.805	0.752	0.511	0.769	0.841	0.133	0.367	0.618	0.091	0.744	0.156	0.599	0.247	0.908	0.263	0.351	0.272	0.969	^*****^

**Table 3:** **Partition of the molecular variance (AMOVA) among and within pine seedling groups**.

**Source**	**df**	**SS**	**MS**	**Est. Var**.	**%**	***F*- Statistics**	**P(rand ≥data)**
Among populations	19	127.345	6.702	0.153	5	*F*_ST_ = 0.054	0.001
Among individuals	161	632.550	3.929	1.234	43	*F*_IS_ = 0.458	0.001
Within individuals	181	264.500	1.461	1.461	51	*F*_IT_ = 0.487	0.001
Total	361	1024.395		2.848	100		

*Probability, P(rand ≥ data), for F_ST_, F_IS_ and F_IT_ is based on permutation across the full data set*.

**Figure 2:**
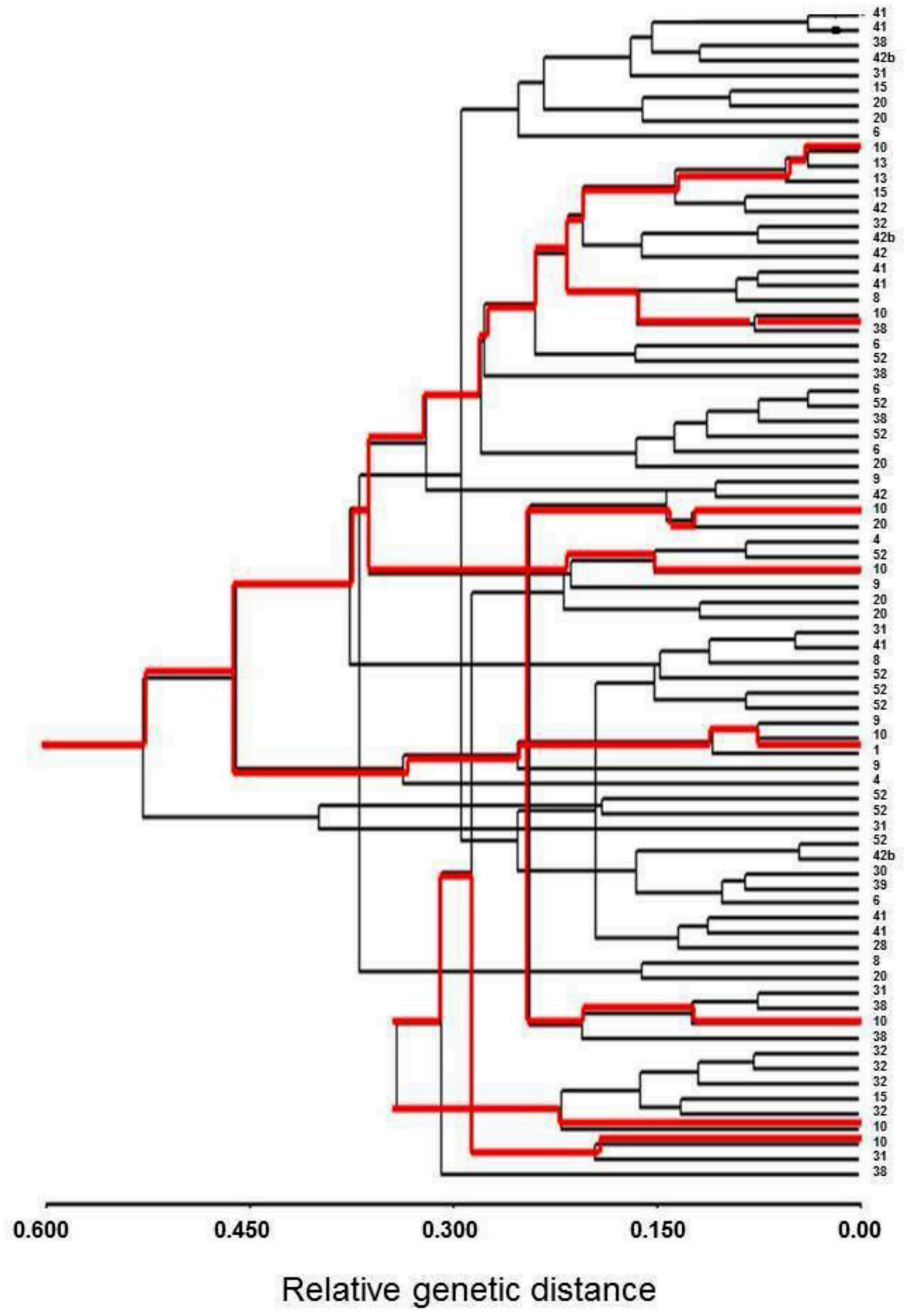
**Cluster analysis (UPGMA) of Lubim seedlings (*n* = 78)**. The numbers on the right present the seedling group number; the red lines show eight individuals belonging to group #10.

The Bayesian assignment test, STRUCTURE, showed a uniform distribution of the sampled seedling (Figure 2S); this distribution was consistent for all tested number of sub-populations (K) ranging from 2 to 20. Similarly, cluster analysis considering only individual seedlings that generated genotype data for at least 8 microsatellites (*n* = 78), also showed no indication of spatial genetic structure, or a tendency of individuals from the same group to be clustered together (UPGMA analysis, Figure 2), as would have been expected from genetically related individuals.

## Discussion

### Genetic structure of the post-fire pine seedling population

The effect of fire on the genetic structure of new post-fire populations are multifaceted, vary among ecosystems, and are associated with the ecology, mating system and post-fire demography of the focus species (reviewed by Steinitz et al., 2012b). In this study we analyzed the genetic structure of post-fire seedling population of *P. halepensis* as a model for an obligate seeding tree with wind pollination and seed dispersal systems. The fire related traits of this species, post fire regeneration niche and demography are relatively well known (Ne'eman and Trabaud, 2000). The spatial pattern of the post-fire seedling, sapling and adult pines is largely determined by the spatial pattern of the large trees of pre-fire forest (Ne'eman and Izhaki, 1998). Because the distance for seed dispersal is limited and gene exchange is probably little, the post-fire genetic structure and diversity of the post-fire seedling generation is also expected to be similar to that of the pre-fire pine generation. Indeed, supporting our first hypothesis, the observed heterozygosity level (Ho = 0.45) of the post-fire seedling population at Lubim was similar to that found in other three nearby populations on Mt. Carmel, Israel (Table 2S). Using comparable microsatellite analyses, our studies revealed that the genetic diversity of the *P. halepensis* populations found on Mt. Carmel was higher than that found in other pine forests in Israel, where heterozygosity levels of 0.35 were found in natural unburned pine forests (Steinitz, 2010), and 0.2–0.25 in a naturally expanding *P. halepensis* population (Troupin et al., 2006).

The post-fire seedling population in Lubim exhibited Hardy-Weinberg equilibrium, indicating a random mating system in the pre-fire generation, and an inbreeding coefficient (*F*_IS_) that was not significantly different from zero, as was also found for other unburned populations in the Carmel (Table 2S). These results differ from previous findings in which several natural populations of *P. halepensis* in Israel indicated a certain level of inbreeding (i.e., positive *F*_IS_ values; Troupin et al., 2006; Steinitz, 2010; Steinitz et al., 2012a). Moreover, a considerable selfing rate of 26–43% was recently found for the *P. halepensis* population on Mt. Carmel (using the same set of microsatellite; Shohami and Nathan, 2014).

Genetic differentiation between native populations at the adult-tree stage indicated a high level of differentiation (*F*_ST_ = 0.32) (Troupin et al., 2006; Steinitz et al., 2012a). Spatial genetic structure was detected in three post-fire (>30 years after fire) populations of *P. clausa*, but not in neighboring older populations (Parker et al., 2001). To the best of our knowledge spatial genetic structure was never examined before in post-fire regenerating seedling populations (i.e., 1–2 years after the fire). In contrast to our second hypothesis, we found no spatial structure within the post-fire tallest seedling population; no correlation was found between genetic and geographic distances (Mantel test). AMOVA results indicated that most of the variation in the genetic composition was due to genetic differences within the groups and not among them.

In the absence of fire, *P. brutia* populations in Greece exhibited high within population genetic diversity compared to that of among populations (Panetsos et al., 1998), and similar partition of genetic diversity was found also in other tree species using allozyme diversity (Hamrick, 2004).

The distances between the seedlings in our study site ranged from few centimeters to about 170 m. Effective seed dispersal distances (without fire) of *P. halepensis* leading to germination and establishment of progeny is 20–70 m from the seed source tree, where most of the offspring did not germinate next to their mothers (Troupin et al., 2006). Parental analyses of a naturally expanding population of *P. halepensis* in the Judean Mountains, Israel, revealed longer effective seed dispersal distance of 6–492 m with an average of 42 m from the mother trees (Steinitz et al., 2011). Such effective seed dispersal distances can eliminate FSSGS formation in a scale of tens of meters.

Seed dispersal distance after fire should not be shorter than that in the absence of fire. The destruction of dense living pine canopies by fire creates two factors that have a positive effect on post-fire dispersal distances in pine samaras: wind speed in the burned forest at canopy height is higher than in the unburned forest, and the absence of the physical obstruction of a dense canopy increases the distance of seed dispersal (Shohami and Nathan, 2014). Once this distance equals the average distance between neighboring trees, it increases random distribution of seed genotypes on the burned forest floor and of the first post-fire seedling generation. In such a case no FSSGS is expected, and this is probably also the explanation of our results.

### The genetic structure of the post-fire seedling groups

Our results implied that most post-fire seedling groups, growing under the same burned tree, originated from more than one mother tree, and in contrast to our third hypothesis, we found no evidence that the dominant seedlings in the pine's favored regeneration niche are siblings, or have a higher rate of genetic similarity. Moreover, although the inbreeding coefficients of the entire post-fire seedling population were close to zero, an excess of heterozygotes and negative inbreeding coefficients in all tested microsatellite (i.e., Ho>He) were found in most of the post-fire seedling groups, implying a general genomic phenomenon. Selective pressure that favors heterozygote individuals at the germination or establishment stages (Hufford and Hamrick, 2003) can explain the apparent excess of heterozygotes among the tallest dominant seedlings.

Our seedlings were not randomly sampled; we have collected the tallest saplings growing under each burned tree, assuming that they reflect advantageous and have high probability to replace the large burned tree. Our results suggest a heterozygote advantage of the post-fire seedling generation in the regeneration niche under the burned canopies of large pine trees. The high pH of the ash and its underlying soil create harsh conditions for the germination of seeds in their regeneration niche (Henig-Sever et al., 1996), which may cause natural selection to favor more vigorous heterozygous individuals. Alternatively, competition between siblings can be stronger than the competition between non-siblings while competing over the same regeneration niche. In such a case, even if most of the seeds that reach the ground under any given large burned pine tree are its offspring, the few that are not can have an advantage, and consequently become the largest saplings in that micro-site.

The wide-ranging excess of heterozygotes found in this research was of noncoding genetic markers (i.e., microsatellites), thus highlighting the general phenomenon of the entire genome of the post-fire seedling group. The strict criteria for outlining balancing selection or heterozygote advantage selection put forward by Hedrick (2012) cannot be applied to our findings, as we cannot specify the fitness of any specific allele or gene. Nonetheless, the significant excess of heterozygotes and their possible adaptive nature cannot be overlooked.

To conclude, the effects of fire on population genetic structure are complex. Our results indicate that the seedling groups growing in their favored regeneration niche under large burned trees are not entirely their sole offspring. The effective seed dispersal distances are probably larger than expected in the post-fire environment, overruling the expected spatial genetic structure. It is likely that intense natural selection pressure in the post-fire regeneration niche causes excess of heterozygous and more vigorous individuals. The mode of the selection is not yet clear, and further studies are needed for revealing its significance. Additional research is required also to further analyze the spatial genetic structure of the developing seedling generation.

## Data archiving statement

Data available from the Dryad Digital Repository: http://dx.doi.org/10.5061/dryad.6r725

### Conflict of interest statement

The authors declare that the research was conducted in the absence of any commercial or financial relationships that could be construed as a potential conflict of interest.
