# Supplementary material for: Genetic Structure of a Naturally Regenerating Post-Fire Seedling Population: Pinus halepensis As a Case Study
Source: Front Plant Sci. 2016 Apr 27;7:549. doi: 10.3389/fpls.2016.00549 (PMC4847172; doi:10.3389/fpls.2016.00549)
Supplement: Supplementary file 1 [file DataSheet1.docx]

**Table 1S:**

Amplification procedure of the microsatellite loci used in the analysis.

| **Reference** | **Accession No.** | **Primer dye** | **MgCl_2_ (mM)** | **Ta (℃)** | **PCR program** | **Locus** |  |
| --- | --- | --- | --- | --- | --- | --- | --- |
| (Guevara et al. 2005) | CR354476 | 6-Fam | 2.5 | 50 | A | A5A12 | |
| (Guevara et al. 2005) | CR377943 | 6-Fam | 1.5 | 50 | A | B4F08 | |
| (González-Martínez et al. 2004) | AY304043 | Vic | 2.5 | 50 | A | PtTx3107 | |
| (Steinitz et al. 2011) | JF803641 | Vic | 2.5 | 50 | A | pEST2669 | |
| (González-Martínez et al. 2004) | AY304036 | 6-Fam | 4.5 | 50 | A | PtTX3116 | |
| (Steinitz et al. 2012) | -- | Ned | 3.5 | 54 | A | pEST1489 | |
| (Keys et al. 2000) | AF195536 | Pet | 2.5 | 50-60 | B | PHAF2 | |
| (Mariette et al. 2001) | AJ012087 | Pet | 2.5 | 59 | A | ITPH4516 | |
| (Keys et al. 2000) | AF195535 | 6-Fam | 2.5 | 56 | C | PHAF01 | |

PCR programs: (**A**) 95°C for 4 minutes; 35 amplification cycles when each cycle is 94°C for 30 sec, annealing temperature (Ta) for 30 sec and 72°C for 45 sec; following by extension of 72°C for 10 minutes. (**B**) 95°C for 5 minutes; 94°C for 30 seconds; 10 touch-down cycles: temperature decrease each cycle by 1^o^C, starting from 60°C and ending with 51°C followed by 30 amplification cycles of 94°C for 30 seconds, 50°C for 30 seconds and 72°C for 90 seconds; extension of 72°C for 8 minutes. (**C**) 95°C for 5 minutes; 35 amplification cycles of 94°C for 90 seconds, 56°C for 90 seconds, 72°C for 90 seconds; extension of 72°C for 10 minutes.

**Table 2S:**

**Genetic diversity of Mt Carmel pine populations tested by the same nine microsatellites. Ho – observes heterozygosity; He unbiased expected heterozygosity; F - Fixation Index = (He - Ho) / He.**

| **Population** |  | **N** | **Ho** | **He** | **F** |
| --- | --- | --- | --- | --- | --- |
| **Lubim*** | **Mean** | 181 | 0.450 | 0.444 | -0.018 |
|  | **SE** |  | 0.080 | 0.079 | 0.025 |
|  |  |  |  |  |  |
| **Mitla*** | **Mean** | 42 | 0.450 | 0.444 | -0.010 |
|  | **SE** |  | 0.083 | 0.076 | 0.047 |
|  |  |  |  |  |  |
| **Antena** | **Mean** | 33 | 0.479 | 0.461 | -0.060 |
|  | **SE** |  | 0.078 | 0.075 | 0.051 |
|  |  |  |  |  |  |
| **Beit Oren** | **Mean** | 31 | 0.413 | 0.437 | 0.062 |
|  | **SE** |  | 0.092 | 0.077 | 0.084 |

*** Populations that have a fire history Table 3S:**

**Inbreeding coefficient within and among the groups**

|  | ***F*is** | ***F*it** |  |
| --- | --- | --- | --- |
| **Mean** | -0.149 | 0.017 |  |
| **SE** | 0.028 | 0.043 |  |

**Table 4S:**

**Chi Square tests for Hardy-Weinberg equilibrium of different loci within Lubim population. Non-significant result (NS) indicates the existence of Hardy-Weinberg equilibrium.**

| **Locus** | **ChiSquare** | **DF** | **Prob** |  |
| --- | --- | --- | --- | --- |
| **A5A12** | 0.062 | 1 | 0.804 | **ns** |
| **B4F08** | 0.640 | 3 | 0.887 | **ns** |
| **PtTx3107** | 0.194 | 1 | 0.659 | **ns** |
| **Pest2669** | 2.250 | 3 | 0.522 | **ns** |
| **PtTx3116** | 0.031 | 1 | 0.860 | **ns** |
| **pEST1489** | 0.130 | 1 | 0.719 | **ns** |
| **PHAF2** | 1.111 | 1 | 0.292 | **ns** |
| **ITPH4516** | 2.619 | 3 | 0.454 | **ns** |
| **PHAF01** | 1.103 | 1 | 0.294 | **ns** |

**Table 5S:**

**Genetic diversity (Ho and unbiased He) and inbreeding coefficient (*F*is) in all tested groups and each loci** (Heterozygote excess was observed in 108 out of 147 tests; *p* < 0.0001, two-tail Sign test).

| **Group** | **Locus** | **N** | **Ho** | **uHe** | **F** |
| --- | --- | --- | --- | --- | --- |
| **1** | **A5A12** | 5 | 0.400 | 0.356 | -0.250 |
|  | **B4F08** | 6 | 1.000 | 0.545 | -1.000 |
|  | **PtTx3107** | 2 | 0.000 | 0.000 | N/A |
|  | **Pest2669** | 6 | 0.667 | 0.485 | -0.500 |
|  | **PtTx3116** | 6 | 0.167 | 0.167 | -0.091 |
|  | **pEST1489** | 0 | 0.000 | 0.000 |  |
|  | **PHAF2** | 7 | 0.429 | 0.363 | -0.273 |
|  | **ITPH4516** | 5 | 0.800 | 0.911 | 0.024 |
|  | **PHAF01** | 6 | 0.500 | 0.530 | -0.029 |
|  |  |  |  |  |  |
| **2** | **A5A12** | 5 | 0.400 | 0.356 | -0.250 |
|  | **B4F08** | 4 | 1.000 | 0.750 | -0.524 |
|  | **PtTx3107** | 5 | 0.200 | 0.467 | 0.524 |
|  | **Pest2669** | 5 | 0.600 | 0.622 | -0.071 |
|  | **PtTx3116** | 4 | 0.250 | 0.250 | -0.143 |
|  | **pEST1489** | 1 | 0.000 | 0.000 | N/A |
|  | **PHAF2** | 9 | 0.111 | 0.111 | -0.059 |
|  | **ITPH4516** | 7 | 0.857 | 0.835 | -0.105 |
|  | **PHAF01** | 7 | 0.714 | 0.582 | -0.321 |
|  |  |  |  |  |  |
| **3** | **A5A12** | 6 | 0.333 | 0.303 | -0.200 |
|  | **B4F08** | 8 | 1.000 | 0.633 | -0.684 |
|  | **PtTx3107** | 7 | 0.286 | 0.440 | 0.300 |
|  | **Pest2669** | 7 | 0.571 | 0.703 | 0.125 |
|  | **PtTx3116** | 7 | 0.000 | 0.000 | N/A |
|  | **pEST1489** | 6 | 0.333 | 0.303 | -0.200 |
|  | **PHAF2** | 9 | 0.556 | 0.425 | -0.385 |
|  | **ITPH4516** | 6 | 0.833 | 0.742 | -0.224 |
|  | **PHAF01** | 8 | 0.625 | 0.592 | -0.127 |
|  |  |  |  |  |  |
| **4** | **A5A12** | 3 | 0.333 | 0.333 | -0.200 |
|  | **B4F08** | 6 | 0.833 | 0.667 | -0.364 |
|  | **PtTx3107** | 5 | 0.200 | 0.200 | -0.111 |
|  | **Pest2669** | 6 | 0.667 | 0.712 | -0.021 |
|  | **PtTx3116** | 4 | 0.000 | 0.000 | N/A |
|  | **pEST1489** | 5 | 0.400 | 0.356 | -0.250 |
|  | **PHAF2** | 6 | 0.667 | 0.485 | -0.500 |
|  | **ITPH4516** | 5 | 1.000 | 0.911 | -0.220 |
|  | **PHAF01** | 7 | 0.714 | 0.495 | -0.556 |
|  |  |  |  |  |  |
| **5** | **A5A12** | 6 | 0.333 | 0.303 | -0.200 |
|  | **B4F08** | 7 | 0.857 | 0.670 | -0.377 |
|  | **PtTx3107** | 5 | 0.400 | 0.356 | -0.250 |
|  | **Pest2669** | 7 | 0.429 | 0.495 | 0.067 |
|  | **PtTx3116** | 9 | 0.000 | 0.000 | N/A |
|  | **pEST1489** | 6 | 0.167 | 0.530 | 0.657 |
|  | **PHAF2** | 8 | 0.625 | 0.525 | -0.270 |
|  | **ITPH4516** | 8 | 0.625 | 0.842 | 0.208 |
|  | **PHAF01** | 8 | 0.500 | 0.592 | 0.099 |
|  |  |  |  |  |  |
| **6** | **A5A12** | 8 | 0.125 | 0.125 | -0.067 |
|  | **B4F08** | 9 | 0.667 | 0.569 | -0.241 |
|  | **PtTx3107** | 8 | 0.375 | 0.325 | -0.231 |
|  | **Pest2669** | 8 | 0.500 | 0.508 | -0.049 |
|  | **PtTx3116** | 9 | 0.000 | 0.000 | N/A |
|  | **pEST1489** | 8 | 0.500 | 0.533 | 0.000 |
|  | **PHAF2** | 9 | 0.444 | 0.569 | 0.172 |
|  | **ITPH4516** | 9 | 0.667 | 0.824 | 0.143 |
|  | **PHAF01** | 8 | 0.250 | 0.492 | 0.458 |
|  |  |  |  |  |  |
| **7** | **A5A12** | 3 | 0.000 | 0.000 | #N/A |
|  | **B4F08** | 8 | 0.750 | 0.500 | -0.600 |
|  | **PtTx3107** | 6 | 0.167 | 0.167 | -0.091 |
|  | **Pest2669** | 6 | 0.167 | 0.167 | -0.091 |
|  | **PtTx3116** | 5 | 0.000 | 0.000 | N/A |
|  | **pEST1489** | 5 | 0.400 | 0.356 | -0.250 |
|  | **PHAF2** | 7 | 0.714 | 0.538 | -0.429 |
|  | **ITPH4516** | 6 | 0.833 | 0.773 | -0.176 |
|  | **PHAF01** | 7 | 0.429 | 0.495 | 0.067 |
|  |  |  |  |  |  |
| **8** | **A5A12** | 3 | 0.000 | 0.000 | N/A |
|  | **B4F08** | 7 | 0.714 | 0.670 | -0.148 |
|  | **PtTx3107** | 4 | 0.250 | 0.536 | 0.467 |
|  | **Pest2669** | 8 | 0.500 | 0.575 | 0.072 |
|  | **PtTx3116** | 7 | 0.000 | 0.000 | N/A |
|  | **pEST1489** | 7 | 0.429 | 0.495 | 0.067 |
|  | **PHAF2** | 8 | 0.625 | 0.458 | -0.455 |
|  | **ITPH4516** | 6 | 0.500 | 0.561 | 0.027 |
|  | **PHAF01** | 7 | 0.714 | 0.538 | -0.429 |
|  |  |  |  |  |  |
| **9** | **A5A12** | 7 | 0.286 | 0.275 | -0.120 |
|  | **B4F08** | 7 | 0.429 | 0.385 | -0.200 |
|  | **PtTx3107** | 6 | 0.000 | 0.000 | N/A |
|  | **Pest2669** | 9 | 1.000 | 0.778 | -0.361 |
|  | **PtTx3116** | 9 | 0.000 | 0.000 | N/A |
|  | **pEST1489** | 6 | 0.833 | 0.530 | -0.714 |
|  | **PHAF2** | 10 | 0.600 | 0.526 | -0.200 |
|  | **ITPH4516** | 7 | 1.000 | 0.857 | -0.256 |
|  | **PHAF01** | 10 | 0.700 | 0.521 | -0.414 |
|  |  |  |  |  |  |
| **10** | **A5A12** | 7 | 0.286 | 0.264 | -0.167 |
|  | **B4F08** | 6 | 0.500 | 0.439 | -0.241 |
|  | **PtTx3107** | 8 | 0.000 | 0.000 | N/A |
|  | **Pest2669** | 8 | 0.750 | 0.708 | -0.129 |
|  | **PtTx3116** | 8 | 0.125 | 0.125 | -0.067 |
|  | **pEST1489** | 5 | 0.400 | 0.533 | 0.167 |
|  | **PHAF2** | 10 | 0.600 | 0.442 | -0.429 |
|  | **ITPH4516** | 7 | 0.857 | 0.758 | -0.217 |
|  | **PHAF01** | 9 | 0.444 | 0.523 | 0.100 |
|  |  |  |  |  |  |
| **11** | **A5A12** | 1 | 0.000 | 0.000 | N/A |
|  | **B4F08** | 3 | 0.000 | 0.533 | 1.000 |
|  | **PtTx3107** | 1 | 0.000 | 0.000 | N/A |
|  | **Pest2669** | 3 | 1.000 | 0.733 | -0.636 |
|  | **PtTx3116** | 5 | 0.000 | 0.000 | N/A |
|  | **pEST1489** | 1 | 0.000 | 0.000 | N/A |
|  | **PHAF2** | 3 | 0.667 | 0.533 | -0.500 |
|  | **ITPH4516** | 1 | 0.000 | 0.000 | N/A |
|  | **PHAF01** | 5 | 0.600 | 0.644 | -0.034 |
|  |  |  |  |  |  |
| **12** | **A5A12** | 6 | 0.167 | 0.167 | -0.091 |
|  | **B4F08** | 6 | 0.333 | 0.621 | 0.415 |
|  | **PtTx3107** | 7 | 0.000 | 0.000 | N/A |
|  | **Pest2669** | 5 | 0.200 | 0.644 | 0.655 |
|  | **PtTx3116** | 6 | 0.000 | 0.000 | N/A |
|  | **pEST1489** | 6 | 1.000 | 0.545 | -1.000 |
|  | **PHAF2** | 8 | 0.125 | 0.125 | -0.067 |
|  | **ITPH4516** | 8 | 0.750 | 0.808 | 0.010 |
|  | **PHAF01** | 8 | 0.375 | 0.458 | 0.127 |
|  |  |  |  |  |  |
| **13** | **A5A12** | 6 | 0.000 | 0.000 | N/A |
|  | **B4F08** | 8 | 0.750 | 0.608 | -0.315 |
|  | **PtTx3107** | 7 | 0.857 | 0.527 | -0.750 |
|  | **Pest2669** | 8 | 0.375 | 0.342 | -0.171 |
|  | **PtTx3116** | 5 | 0.000 | 0.000 | N/A |
|  | **pEST1489** | 5 | 0.200 | 0.200 | -0.111 |
|  | **PHAF2** | 7 | 0.429 | 0.495 | 0.067 |
|  | **ITPH4516** | 7 | 0.857 | 0.868 | -0.063 |
|  | **PHAF01** | 7 | 0.571 | 0.527 | -0.167 |
|  |  |  |  |  |  |
| **14** | **A5A12** | 8 | 0.250 | 0.233 | -0.143 |
|  | **B4F08** | 9 | 0.556 | 0.451 | -0.304 |
|  | **PtTx3107** | 9 | 0.444 | 0.366 | -0.286 |
|  | **Pest2669** | 8 | 0.875 | 0.617 | -0.514 |
|  | **PtTx3116** | 8 | 0.000 | 0.000 | N/A |
|  | **pEST1489** | 7 | 0.429 | 0.495 | 0.067 |
|  | **PHAF2** | 8 | 0.375 | 0.458 | 0.127 |
|  | **ITPH4516** | 7 | 1.000 | 0.857 | -0.256 |
|  | **PHAF01** | 9 | 0.444 | 0.608 | 0.226 |
|  |  |  |  |  |  |
| **15** | **A5A12** | 2 | 0.000 | 0.000 | N/A |
|  | **B4F08** | 3 | 1.000 | 0.600 | -1.000 |
|  | **PtTx3107** | 2 | 0.500 | 0.500 | -0.333 |
|  | **Pest2669** | 5 | 1.000 | 0.644 | -0.724 |
|  | **PtTx3116** | 4 | 0.250 | 0.250 | -0.143 |
|  | **pEST1489** | 2 | 1.000 | 0.667 | -1.000 |
|  | **PHAF2** | 5 | 0.000 | 0.000 | N/A |
|  | **ITPH4516** | 3 | 0.667 | 0.867 | 0.077 |
|  | **PHAF01** | 4 | 0.750 | 0.679 | -0.263 |
|  |  |  |  |  |  |
| **16** | **A5A12** | 8 | 0.125 | 0.125 | -0.067 |
|  | **B4F08** | 7 | 0.714 | 0.670 | -0.148 |
|  | **PtTx3107** | 7 | 0.143 | 0.143 | -0.077 |
|  | **Pest2669** | 8 | 0.750 | 0.667 | -0.200 |
|  | **PtTx3116** | 9 | 0.111 | 0.111 | -0.059 |
|  | **pEST1489** | 7 | 0.429 | 0.538 | 0.143 |
|  | **PHAF2** | 8 | 0.250 | 0.233 | -0.143 |
|  | **ITPH4516** | 7 | 1.000 | 0.813 | -0.324 |
|  | **PHAF01** | 9 | 0.556 | 0.503 | -0.169 |
|  |  |  |  |  |  |
| **17** | **A5A12** | 5 | 0.000 | 0.000 | N/A |
|  | **B4F08** | 6 | 0.667 | 0.621 | -0.171 |
|  | **PtTx3107** | 4 | 0.250 | 0.250 | -0.143 |
|  | **Pest2669** | 6 | 0.333 | 0.318 | -0.143 |
|  | **PtTx3116** | 3 | 0.000 | 0.000 | N/A |
|  | **pEST1489** | 5 | 0.600 | 0.556 | -0.200 |
|  | **PHAF2** | 8 | 0.750 | 0.500 | -0.600 |
|  | **ITPH4516** | 7 | 0.714 | 0.714 | -0.077 |
|  | **PHAF01** | 3 | 0.333 | 0.333 | -0.200 |
|  |  |  |  |  |  |
| **18** | **A5A12** | 8 | 0.625 | 0.525 | -0.270 |
|  | **B4F08** | 9 | 0.778 | 0.601 | -0.370 |
|  | **PtTx3107** | 9 | 0.111 | 0.111 | -0.059 |
|  | **Pest2669** | 7 | 0.429 | 0.604 | 0.236 |
|  | **PtTx3116** | 7 | 0.000 | 0.000 | N/A |
|  | **pEST1489** | 8 | 0.375 | 0.458 | 0.127 |
|  | **PHAF2** | 9 | 0.222 | 0.366 | 0.357 |
|  | **ITPH4516** | 9 | 0.778 | 0.765 | -0.077 |
|  | **PHAF01** | 9 | 0.444 | 0.503 | 0.065 |
|  |  |  |  |  |  |
| **19** | **A5A12** | 5 | 0.200 | 0.200 | -0.111 |
|  | **B4F08** | 7 | 1.000 | 0.648 | -0.661 |
|  | **PtTx3107** | 7 | 0.286 | 0.440 | 0.300 |
|  | **Pest2669** | 7 | 0.286 | 0.582 | 0.472 |
|  | **PtTx3116** | 4 | 0.000 | 0.000 | N/A |
|  | **pEST1489** | 1 | 0.000 | 0.000 | N/A |
|  | **PHAF2** | 9 | 0.444 | 0.366 | -0.286 |
|  | **ITPH4516** | 8 | 0.625 | 0.792 | 0.158 |
|  | **PHAF01** | 8 | 0.375 | 0.458 | 0.127 |
|  |  |  |  |  |  |
| **20** | **A5A12** | 5 | 0.200 | 0.200 | -0.111 |
|  | **B4F08** | 8 | 0.500 | 0.542 | 0.015 |
|  | **PtTx3107** | 7 | 0.286 | 0.264 | -0.167 |
|  | **Pest2669** | 9 | 0.667 | 0.529 | -0.333 |
|  | **PtTx3116** | 4 | 0.000 | 0.000 | N/A |
|  | **pEST1489** | 1 | 0.000 | 0.000 | N/A |
|  | **PHAF2** | 10 | 0.500 | 0.395 | -0.333 |
|  | **ITPH4516** | 7 | 0.714 | 0.670 | -0.148 |
|  | **PHAF01** | 9 | 0.667 | 0.523 | -0.350 |

**Table 6S:**

**ITPH4516 microsatellite genotypes of all seedlings, number of alleles and their size (in bp) by groups, and the alleles of the possible groups’ single mothers.**

| **ID** | **Group** | **ITPH4516**  **alleles (bp)** | | **No. of alleles** | **Alleles of the possible**  **groups’ single mothers** |
| --- | --- | --- | --- | --- | --- |
| 1-03 | 1 | 140 | 154 | 7 | More than one mother |
| 1-04 | 1 | 142 | 166 |  |  |
| 1-05 | 1 | 158 | 168 |  |  |
| 1-06 | 1 | 140 | 140 |  |  |
| 1-07 | 1 | 158 | 160 |  |  |
| 4-02 | 4 | 158 | 176 | 6 | More than one mother |
| 4-03 | 4 | 158 | 158 |  |  |
| 4-06 | 4 | 140 | 176 |  |  |
| 4-07 | 4 | 140 | 160 |  |  |
| 4-08 | 4 | 140 | 164 |  |  |
| 4-09 | 4 | 140 | 158 |  |  |
| 4-10 | 4 | 154 | 176 |  |  |
| 6-02 | 6 | 140 | 158 | 5 | 140/158 |
| 6-03 | 6 | 158 | 168 |  |  |
| 6-04 | 6 | 140 | 164 |  |  |
| 6-06 | 6 | 142 | 158 |  |  |
| 6-07 | 6 | 158 | 168 |  |  |
| 6-09 | 6 | 158 | 158 |  |  |
| 8-02 | 8 | 140 | 142 | 7 | More than one mother |
| 8-03 | 8 | 160 | 176 |  |  |
| 8-04 | 8 | 140 | 158 |  |  |
| 8-07 | 8 | 136 | 158 |  |  |
| 8-09 | 8 | 158 | 166 |  |  |
| 9-01 | 9 | 140 | 140 | 6 | More than one mother |
| 9-02 | 9 | 170 | 176 |  |  |
| 9-03 | 9 | 176 | 176 |  |  |
| 9-04 | 9 | 140 | 158 |  |  |
| 9-06 | 9 | 140 | 164 |  |  |
| 9-07 | 9 | 136 | 176 |  |  |
| 9-08 | 9 | 140 | 168 |  |  |
| 9-09 | 9 | 164 | 164 |  |  |
| 10-01 | 10 | 140 | 164 | 6 | More than one mother |
| 10-02 | 10 | 158 | 176 |  |  |
| 10-04 | 10 | 140 | 158 |  |  |
| 10-05 | 10 | 140 | 140 |  |  |
| 10-06 | 10 | 136 | 140 |  |  |
| 10-07 | 10 | 164 | 164 |  |  |
| 10-08 | 10 | 140 | 158 |  |  |
| 10-09 | 10 | 142 | 176 |  |  |
| 10-10 | 10 | 176 | 176 |  |  |
| 13-01 | 13 | 140 | 176 | 4 | More than one mother |
| 13-02 | 13 | 158 | 158 |  |  |
| 13-03 | 13 | 158 | 176 |  |  |
| 13-05 | 13 | 158 | 176 |  |  |
| 13-08 | 13 | 140 | 142 |  |  |
| 13-09 | 13 | 140 | 176 |  |  |
| 15-01 | 15 | 158 | 158 | 4 | 158/176 |
| 15-02 | 15 | 158 | 176 |  |  |
| 15-04 | 15 | 140 | 158 |  |  |
| 15-05 | 15 | 142 | 176 |  |  |
| 15-08 | 15 | 158 | 158 |  |  |
| 15-09 | 15 | 158 | 158 |  |  |
| 20-01 | 20 | 170 | 176 | 8 |  |
| 20-03 | 20 | 140 | 158 |  | More than one mother |
| 20-04 | 20 | 140 | 154 |  |  |
| 20-07 | 20 | 136 | 140 |  |  |
| 20-08 | 20 | 140 | 168 |  |  |
| 20-09 | 20 | 158 | 162 |  |  |
| 20-10 | 20 | 140 | 158 |  |  |
| 21-01 | 21 | 136 | 140 | 5 | 136/158 |
| 21-03 | 21 | 158 | 158 |  |  |
| 21-05 | 21 | 136 | 158 |  |  |
| 21-06 | 21 | 136 | 160 |  |  |
| 21-08 | 21 | 158 | 160 |  |  |
| 21-09 | 21 | 158 | 174 |  |  |
| 21-10 | 21 | 136 | 158 |  |  |
| 28-07 | 28 | 158 | 158 | 1 | 158/??? |
| 31-01 | 31 | 140 | 158 | 6 | 140/176 |
| 31-02 | 31 | 168 | 176 |  |  |
| 31-03 | 31 | 140 | 140 |  |  |
| 31-05 | 31 | 140 | 162 |  |  |
| 31-06 | 31 | 168 | 176 |  |  |
| 31-07 | 31 | 176 | 176 |  |  |
| 31-08 | 31 | 140 | 172 |  |  |
| 31-10 | 31 | 140 | 158 |  |  |
| 32-02 | 32 | 142 | 176 | 6 | More than one mother |
| 32-03 | 32 | 140 | 158 |  |  |
| 32-04 | 32 | 164 | 176 |  |  |
| 32-05 | 32 | 140 | 160 |  |  |
| 32-06 | 32 | 164 | 176 |  |  |
| 32-09 | 32 | 142 | 158 |  |  |
| 32-10 | 32 | 140 | 140 |  |  |
| 38-01 | 38 | 140 | 160 | 6 | More than one mother |
| 38-02 | 38 | 140 | 174 |  |  |
| 38-04 | 38 | 140 | 158 |  |  |
| 38-05 | 38 | 140 | 158 |  |  |
| 38-07 | 38 | 158 | 174 |  |  |
| 38-08 | 38 | 170 | 174 |  |  |
| 38-09 | 38 | 138 | 160 |  |  |
| 39-02 | 39 | 142 | 158 | 4 | 140/158 |
| 39-05 | 39 | 140 | 140 |  |  |
| 39-10 | 39 | 158 | 164 |  |  |
| 41-01 | 41 | 158 | 160 | 7 | 138/158 or 154/158 |
| 41-02 | 41 | 158 | 170 |  |  |
| 41-03 | 41 | 158 | 164 |  |  |
| 41-04 | 41 | 140 | 158 |  |  |
| 41-07 | 41 | 138 | 154 |  |  |
| 41-08 | 41 | 140 | 158 |  |  |
| 41-09 | 41 | 158 | 164 |  |  |
| 42-01 | 42 | 140 | 176 | 4 | 140/158 |
| 42-02 | 42 | 140 | 140 |  |  |
| 42-04 | 42 | 140 | 158 |  |  |
| 42-06 | 42 | 158 | 176 |  |  |
| 42-07 | 42 | 158 | 158 |  |  |
| 42-08 | 42 | 140 | 158 |  |  |
| 42-09 | 42 | 140 | 160 |  |  |
| 52-01 | 52 | 158 | 160 | 7 | More than one mother |
| 52-02 | 52 | 158 | 164 |  |  |
| 52-03 | 52 | 170 | 176 |  |  |
| 52-04 | 52 | 158 | 164 |  |  |
| 52-05 | 52 | 140 | 164 |  |  |
| 52-06 | 52 | 164 | 164 |  |  |
| 52-07 | 52 | 158 | 164 |  |  |
| 52-08 | 52 | 158 | 158 |  |  |
| 52-09 | 52 | 164 | 168 |  |  |
| 77-01 | 77 | 140 | 140 | 5 | More than one mother |
| 77-02 | 77 | 140 | 158 |  |  |
| 77-03 | 77 | 158 | 158 |  |  |
| 77-05 | 77 | 158 | 164 |  |  |
| 77-06 | 77 | 158 | 168 |  |  |
| 77-07 | 77 | 158 | 168 |  |  |
| 77-09 | 77 | 140 | 174 |  |  |
| 77-10 | 77 | 164 | 164 |  |  |
| 42b-01 | 42b | 158 | 164 | 3 | 140/158 |
| 42b-03 | 42b | 140 | 164 |  |  |
| 42b-04 | 42b | 140 | 140 |  |  |
| 42b-05 | 42b | 158 | 164 |  |  |
| 42b-06 | 42b | 140 | 164 |  |  |
| 42b-08 | 42b | 140 | 140 |  |  |
| 42b-09 | 42b | 140 | 158 |  |  |

**Table 7S:**

**Pairwise population Fst values and estimates of number of migrants (Nm) based on Nei Unbiased Genetic Distance**

| **Group 1** | **Group 2** | **Fst** | **Nm** | **#Group 1** | **#Group 2** |
| --- | --- | --- | --- | --- | --- |
| Group 1 | Group 2 | 0.149 | 1.427 | 8 | 9 |
| Group 1 | Group 3 | 0.133 | 1.626 | 8 | 10 |
| Group 2 | Group 3 | 0.037 | 6.499 | 9 | 10 |
| Group 1 | Group 4 | 0.112 | 1.987 | 8 | 9 |
| Group 2 | Group 4 | 0.051 | 4.615 | 9 | 9 |
| Group 3 | Group 4 | 0.023 | 10.624 | 10 | 9 |
| Group 1 | Group 5 | 0.130 | 1.675 | 8 | 10 |
| Group 2 | Group 5 | 0.085 | 2.697 | 9 | 10 |
| Group 3 | Group 5 | 0.050 | 4.715 | 10 | 10 |
| Group 4 | Group 5 | 0.049 | 4.837 | 9 | 10 |
| Group 1 | Group 6 | 0.134 | 1.620 | 8 | 10 |
| Group 2 | Group 6 | 0.086 | 2.642 | 9 | 10 |
| Group 3 | Group 6 | 0.048 | 4.930 | 10 | 10 |
| Group 4 | Group 6 | 0.052 | 4.583 | 9 | 10 |
| Group 5 | Group 6 | 0.018 | 13.435 | 10 | 10 |
| Group 1 | Group 7 | 0.157 | 1.341 | 8 | 10 |
| Group 2 | Group 7 | 0.093 | 2.440 | 9 | 10 |
| Group 3 | Group 7 | 0.066 | 3.518 | 10 | 10 |
| Group 4 | Group 7 | 0.056 | 4.182 | 9 | 10 |
| Group 5 | Group 7 | 0.062 | 3.808 | 10 | 10 |
| Group 6 | Group 7 | 0.033 | 7.401 | 10 | 10 |
| Group 1 | Group 8 | 0.151 | 1.407 | 8 | 10 |
| Group 2 | Group 8 | 0.068 | 3.430 | 9 | 10 |
| Group 3 | Group 8 | 0.028 | 8.788 | 10 | 10 |
| Group 4 | Group 8 | 0.048 | 4.996 | 9 | 10 |
| Group 5 | Group 8 | 0.053 | 4.491 | 10 | 10 |
| Group 6 | Group 8 | 0.041 | 5.849 | 10 | 10 |
| Group 7 | Group 8 | 0.062 | 3.786 | 10 | 10 |
| Group 1 | Group 9 | 0.143 | 1.495 | 8 | 10 |
| Group 2 | Group 9 | 0.108 | 2.069 | 9 | 10 |
| Group 3 | Group 9 | 0.057 | 4.169 | 10 | 10 |
| Group 4 | Group 9 | 0.036 | 6.617 | 9 | 10 |
| Group 5 | Group 9 | 0.056 | 4.198 | 10 | 10 |
| Group 6 | Group 9 | 0.052 | 4.584 | 10 | 10 |
| Group 7 | Group 9 | 0.076 | 3.032 | 10 | 10 |
| Group 8 | Group 9 | 0.068 | 3.427 | 10 | 10 |
| Group 1 | Group 10 | 0.140 | 1.535 | 8 | 10 |
| Group 2 | Group 10 | 0.090 | 2.521 | 9 | 10 |
| Group 3 | Group 10 | 0.047 | 5.049 | 10 | 10 |
| Group 4 | Group 10 | 0.035 | 6.851 | 9 | 10 |
| Group 5 | Group 10 | 0.063 | 3.721 | 10 | 10 |
| Group 6 | Group 10 | 0.068 | 3.406 | 10 | 10 |
| Group 7 | Group 10 | 0.095 | 2.384 | 10 | 10 |
| Group 8 | Group 10 | 0.064 | 3.651 | 10 | 10 |
| Group 9 | Group 10 | 0.025 | 9.570 | 10 | 10 |
| Group 1 | Group 11 | 0.220 | 0.888 | 8 | 7 |
| Group 2 | Group 11 | 0.212 | 0.928 | 9 | 7 |
| Group 3 | Group 11 | 0.168 | 1.242 | 10 | 7 |
| Group 4 | Group 11 | 0.161 | 1.307 | 9 | 7 |
| Group 5 | Group 11 | 0.128 | 1.704 | 10 | 7 |
| Group 6 | Group 11 | 0.127 | 1.712 | 10 | 7 |
| Group 7 | Group 11 | 0.192 | 1.054 | 10 | 7 |
| Group 8 | Group 11 | 0.146 | 1.465 | 10 | 7 |
| Group 9 | Group 11 | 0.175 | 1.175 | 10 | 7 |
| Group 10 | Group 11 | 0.153 | 1.387 | 10 | 7 |
| Group 1 | Group 12 | 0.150 | 1.422 | 8 | 8 |
| Group 2 | Group 12 | 0.087 | 2.628 | 9 | 8 |
| Group 3 | Group 12 | 0.076 | 3.022 | 10 | 8 |
| Group 4 | Group 12 | 0.069 | 3.382 | 9 | 8 |
| Group 5 | Group 12 | 0.051 | 4.628 | 10 | 8 |
| Group 6 | Group 12 | 0.044 | 5.390 | 10 | 8 |
| Group 7 | Group 12 | 0.080 | 2.874 | 10 | 8 |
| Group 8 | Group 12 | 0.085 | 2.690 | 10 | 8 |
| Group 9 | Group 12 | 0.079 | 2.908 | 10 | 8 |
| Group 10 | Group 12 | 0.070 | 3.344 | 10 | 8 |
| Group 11 | Group 12 | 0.147 | 1.453 | 7 | 8 |
| Group 1 | Group 13 | 0.162 | 1.290 | 8 | 8 |
| Group 2 | Group 13 | 0.052 | 4.560 | 9 | 8 |
| Group 3 | Group 13 | 0.039 | 6.169 | 10 | 8 |
| Group 4 | Group 13 | 0.050 | 4.788 | 9 | 8 |
| Group 5 | Group 13 | 0.070 | 3.306 | 10 | 8 |
| Group 6 | Group 13 | 0.056 | 4.227 | 10 | 8 |
| Group 7 | Group 13 | 0.052 | 4.566 | 10 | 8 |
| Group 8 | Group 13 | 0.037 | 6.579 | 10 | 8 |
| Group 9 | Group 13 | 0.087 | 2.626 | 10 | 8 |
| Group 10 | Group 13 | 0.096 | 2.363 | 10 | 8 |
| Group 11 | Group 13 | 0.249 | 0.755 | 7 | 8 |
| Group 12 | Group 13 | 0.115 | 1.931 | 8 | 8 |
| Group 1 | Group 14 | 0.125 | 1.754 | 8 | 10 |
| Group 2 | Group 14 | 0.065 | 3.570 | 9 | 10 |
| Group 3 | Group 14 | 0.033 | 7.267 | 10 | 10 |
| Group 4 | Group 14 | 0.039 | 6.199 | 9 | 10 |
| Group 5 | Group 14 | 0.035 | 6.991 | 10 | 10 |
| Group 6 | Group 14 | 0.021 | 11.470 | 10 | 10 |
| Group 7 | Group 14 | 0.050 | 4.781 | 10 | 10 |
| Group 8 | Group 14 | 0.046 | 5.139 | 10 | 10 |
| Group 9 | Group 14 | 0.068 | 3.442 | 10 | 10 |
| Group 10 | Group 14 | 0.071 | 3.263 | 10 | 10 |
| Group 11 | Group 14 | 0.135 | 1.609 | 7 | 10 |
| Group 12 | Group 14 | 0.053 | 4.503 | 8 | 10 |
| Group 13 | Group 14 | 0.059 | 3.983 | 8 | 10 |
| Group 1 | Group 15 | 0.144 | 1.492 | 8 | 6 |
| Group 2 | Group 15 | 0.067 | 3.507 | 9 | 6 |
| Group 3 | Group 15 | 0.058 | 4.043 | 10 | 6 |
| Group 4 | Group 15 | 0.077 | 2.989 | 9 | 6 |
| Group 5 | Group 15 | 0.063 | 3.712 | 10 | 6 |
| Group 6 | Group 15 | 0.051 | 4.648 | 10 | 6 |
| Group 7 | Group 15 | 0.099 | 2.267 | 10 | 6 |
| Group 8 | Group 15 | 0.058 | 4.087 | 10 | 6 |
| Group 9 | Group 15 | 0.089 | 2.549 | 10 | 6 |
| Group 10 | Group 15 | 0.073 | 3.162 | 10 | 6 |
| Group 11 | Group 15 | 0.158 | 1.330 | 7 | 6 |
| Group 12 | Group 15 | 0.047 | 5.074 | 8 | 6 |
| Group 13 | Group 15 | 0.087 | 2.624 | 8 | 6 |
| Group 14 | Group 15 | 0.059 | 3.988 | 10 | 6 |
| Group 1 | Group 16 | 0.131 | 1.659 | 8 | 9 |
| Group 2 | Group 16 | 0.072 | 3.233 | 9 | 9 |
| Group 3 | Group 16 | 0.049 | 4.838 | 10 | 9 |
| Group 4 | Group 16 | 0.049 | 4.886 | 9 | 9 |
| Group 5 | Group 16 | 0.050 | 4.734 | 10 | 9 |
| Group 6 | Group 16 | 0.062 | 3.761 | 10 | 9 |
| Group 7 | Group 16 | 0.100 | 2.243 | 10 | 9 |
| Group 8 | Group 16 | 0.054 | 4.389 | 10 | 9 |
| Group 9 | Group 16 | 0.064 | 3.666 | 10 | 9 |
| Group 10 | Group 16 | 0.039 | 6.101 | 10 | 9 |
| Group 11 | Group 16 | 0.099 | 2.282 | 7 | 9 |
| Group 12 | Group 16 | 0.043 | 5.585 | 8 | 9 |
| Group 13 | Group 16 | 0.096 | 2.342 | 8 | 9 |
| Group 14 | Group 16 | 0.058 | 4.093 | 10 | 9 |
| Group 15 | Group 16 | 0.047 | 5.067 | 6 | 9 |
| Group 1 | Group 17 | 0.166 | 1.259 | 8 | 9 |
| Group 2 | Group 17 | 0.134 | 1.613 | 9 | 9 |
| Group 3 | Group 17 | 0.090 | 2.539 | 10 | 9 |
| Group 4 | Group 17 | 0.083 | 2.766 | 9 | 9 |
| Group 5 | Group 17 | 0.057 | 4.124 | 10 | 9 |
| Group 6 | Group 17 | 0.022 | 11.007 | 10 | 9 |
| Group 7 | Group 17 | 0.034 | 7.139 | 10 | 9 |
| Group 8 | Group 17 | 0.071 | 3.259 | 10 | 9 |
| Group 9 | Group 17 | 0.066 | 3.530 | 10 | 9 |
| Group 10 | Group 17 | 0.100 | 2.241 | 10 | 9 |
| Group 11 | Group 17 | 0.159 | 1.318 | 7 | 9 |
| Group 12 | Group 17 | 0.085 | 2.697 | 8 | 9 |
| Group 13 | Group 17 | 0.089 | 2.548 | 8 | 9 |
| Group 14 | Group 17 | 0.052 | 4.564 | 10 | 9 |
| Group 15 | Group 17 | 0.101 | 2.214 | 6 | 9 |
| Group 16 | Group 17 | 0.105 | 2.133 | 9 | 9 |
| Group 1 | Group 18 | 0.127 | 1.716 | 8 | 9 |
| Group 2 | Group 18 | 0.069 | 3.357 | 9 | 9 |
| Group 3 | Group 18 | 0.042 | 5.769 | 10 | 9 |
| Group 4 | Group 18 | 0.038 | 6.271 | 9 | 9 |
| Group 5 | Group 18 | 0.054 | 4.366 | 10 | 9 |
| Group 6 | Group 18 | 0.080 | 2.892 | 10 | 9 |
| Group 7 | Group 18 | 0.109 | 2.053 | 10 | 9 |
| Group 8 | Group 18 | 0.082 | 2.785 | 10 | 9 |
| Group 9 | Group 18 | 0.063 | 3.732 | 10 | 9 |
| Group 10 | Group 18 | 0.040 | 6.064 | 10 | 9 |
| Group 11 | Group 18 | 0.183 | 1.114 | 7 | 9 |
| Group 12 | Group 18 | 0.076 | 3.052 | 8 | 9 |
| Group 13 | Group 18 | 0.100 | 2.241 | 8 | 9 |
| Group 14 | Group 18 | 0.073 | 3.157 | 10 | 9 |
| Group 15 | Group 18 | 0.080 | 2.860 | 6 | 9 |
| Group 16 | Group 18 | 0.049 | 4.880 | 9 | 9 |
| Group 17 | Group 18 | 0.136 | 1.591 | 9 | 9 |
| Group 1 | Group 19 | 0.144 | 1.486 | 8 | 9 |
| Group 2 | Group 19 | 0.027 | 9.010 | 9 | 9 |
| Group 3 | Group 19 | 0.022 | 11.056 | 10 | 9 |
| Group 4 | Group 19 | 0.036 | 6.675 | 9 | 9 |
| Group 5 | Group 19 | 0.086 | 2.643 | 10 | 9 |
| Group 6 | Group 19 | 0.082 | 2.795 | 10 | 9 |
| Group 7 | Group 19 | 0.085 | 2.689 | 10 | 9 |
| Group 8 | Group 19 | 0.053 | 4.456 | 10 | 9 |
| Group 9 | Group 19 | 0.090 | 2.528 | 10 | 9 |
| Group 10 | Group 19 | 0.080 | 2.859 | 10 | 9 |
| Group 11 | Group 19 | 0.214 | 0.920 | 7 | 9 |
| Group 12 | Group 19 | 0.112 | 1.973 | 8 | 9 |
| Group 13 | Group 19 | 0.034 | 7.064 | 8 | 9 |
| Group 14 | Group 19 | 0.058 | 4.088 | 10 | 9 |
| Group 15 | Group 19 | 0.084 | 2.718 | 6 | 9 |
| Group 16 | Group 19 | 0.076 | 3.058 | 9 | 9 |
| Group 17 | Group 19 | 0.126 | 1.739 | 9 | 9 |
| Group 18 | Group 19 | 0.072 | 3.226 | 9 | 9 |
| Group 1 | Group 20 | 0.142 | 1.506 | 8 | 10 |
| Group 2 | Group 20 | 0.144 | 1.483 | 9 | 10 |
| Group 3 | Group 20 | 0.112 | 1.977 | 10 | 10 |
| Group 4 | Group 20 | 0.105 | 2.120 | 9 | 10 |
| Group 5 | Group 20 | 0.059 | 3.982 | 10 | 10 |
| Group 6 | Group 20 | 0.067 | 3.474 | 10 | 10 |
| Group 7 | Group 20 | 0.134 | 1.610 | 10 | 10 |
| Group 8 | Group 20 | 0.100 | 2.245 | 10 | 10 |
| Group 9 | Group 20 | 0.089 | 2.569 | 10 | 10 |
| Group 10 | Group 20 | 0.093 | 2.447 | 10 | 10 |
| Group 11 | Group 20 | 0.121 | 1.813 | 7 | 10 |
| Group 12 | Group 20 | 0.094 | 2.417 | 8 | 10 |
| Group 13 | Group 20 | 0.132 | 1.644 | 8 | 10 |
| Group 14 | Group 20 | 0.095 | 2.381 | 10 | 10 |
| Group 15 | Group 20 | 0.081 | 2.824 | 6 | 10 |
| Group 16 | Group 20 | 0.081 | 2.818 | 9 | 10 |
| Group 17 | Group 20 | 0.102 | 2.190 | 9 | 10 |
| Group 18 | Group 20 | 0.109 | 2.039 | 9 | 10 |
| Group 19 | Group 20 | 0.141 | 1.524 | 9 | 10 |


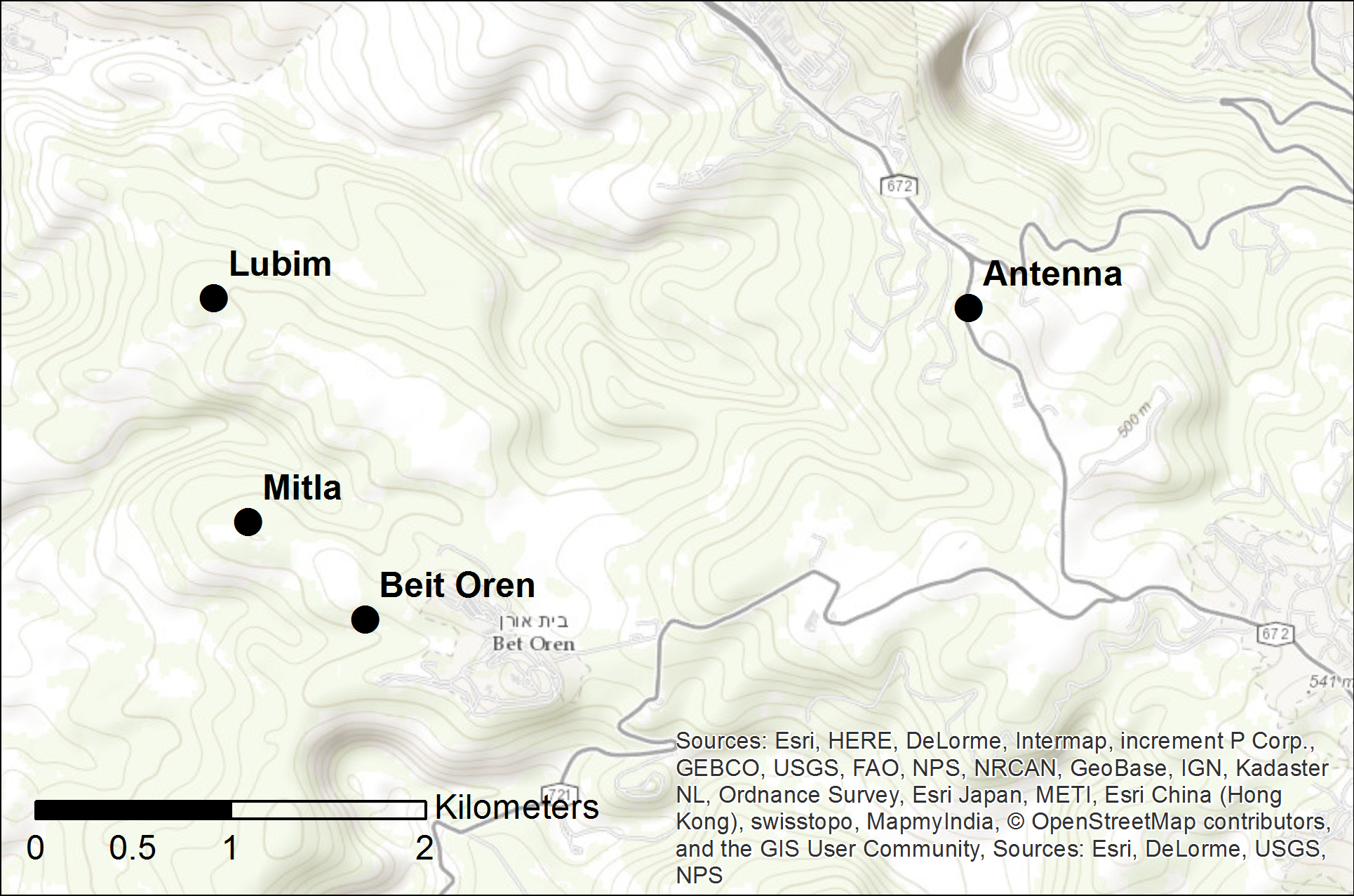


**Figure S1.**

An aerial map of sampling sites


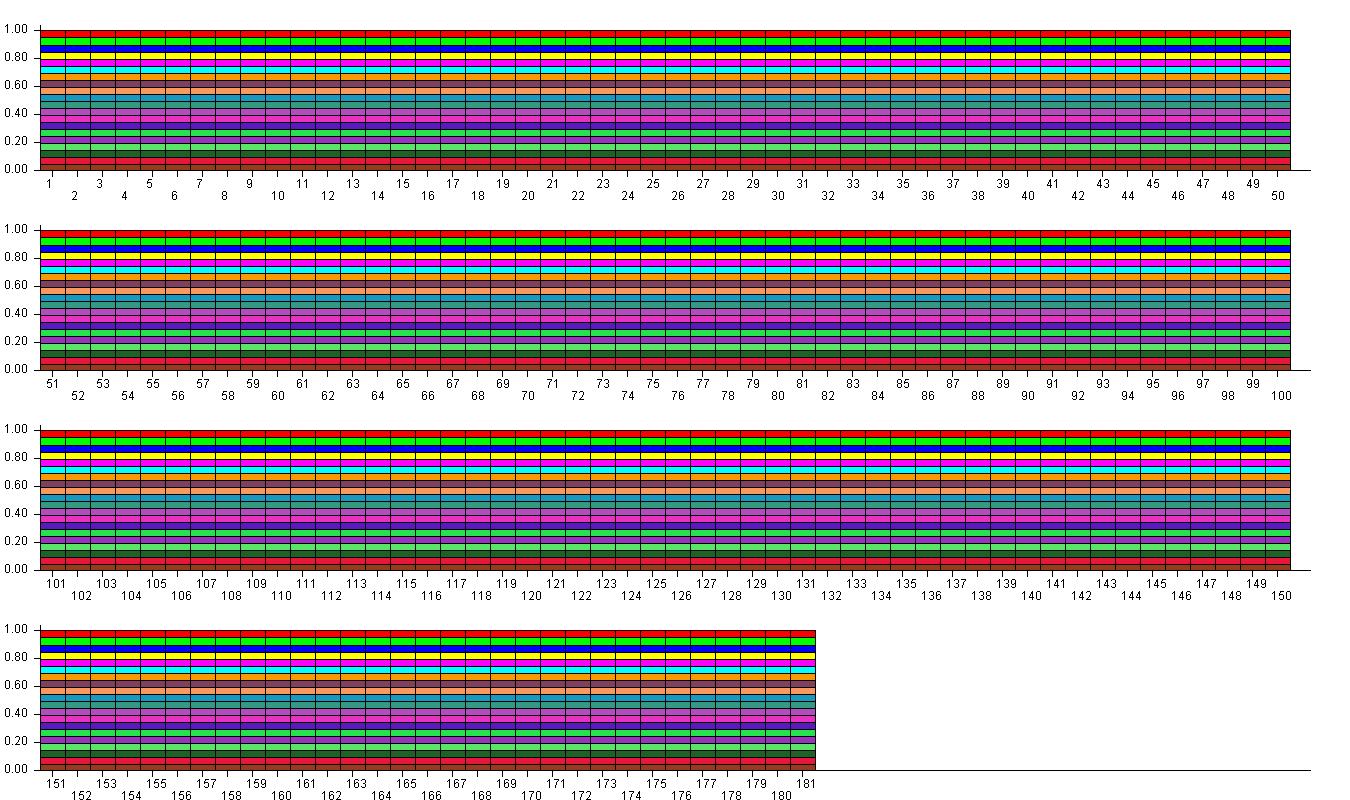


Figure S2.

**STRUCTURE results for division of the post fire seedlings in Lubim to K=20 subpopulation, X-axis represents the individuals analyzed (n=181). The Y axis represent the assigned proportion of each individual to K=20**
